# Case Report: PCI of the left coronary artery after salvage operation in a comatose patient with an acute type A aortic dissection

**DOI:** 10.3389/fcvm.2025.1516152

**Published:** 2025-02-06

**Authors:** Jan Bidovec, Petar Risteski, Barbara E. Stähli, Omer Dzemali, Michael Hofmann, Julia Stehli

**Affiliations:** ^1^ Department of Cardiac Surgery, University Hospital Zurich, Zurich, Switzerland; ^2^ Department of Cardiac Surgery, City Hospital Triemli, Zurich, Switzerland; ^3^ Department of Cardiology, University Hospital Zurich, Zurich, Switzerland

**Keywords:** aortic dissection, cerebral malperfusion, carotid obstruction, left main stenting, coronary artery dissection

## Abstract

A 74-year-old female was found hemiplegic in a public restroom. After arrival in our stroke unit, a computed tomography (CT) was performed, and she was diagnosed with bilateral carotid artery and right vertebral artery occlusion due to an acute type A aortic dissection. The patient deteriorated quickly to a GCS of 3 and was brought to the operating room, where a salvage replacement of the ascending aorta and the proximal aortic arch was performed with unilateral antegrade cerebral perfusion. Three days later, a significant decrease in left ventricular function and increase in cardiac biomarkers were observed. Coronary CT displayed residual dissection of the aortic root, extending into the left main coronary artery. The patient underwent an intravascular ultrasound-guided stenting of the left main, resulting in total recovery of heart function. She was extubated on the fourth postoperative day, with no residual neurological impairment. This case report advocates for the proper management of patients with ATAAD with severe neurological impairment, emphasizing the importance of a robust multidisciplinary approach in their care.

## 1 Introduction

Preoperative cerebral malperfusion in patients with an acute type A aortic dissection (ATAAD) is associated with poor postoperative outcomes. The prognosis of comatose patients with bilateral carotid artery obstruction and last-remaining-vessel cerebral perfusion is particularly desolate (1–3). The International Registry of Acute Aortic Dissection (IRAD) investigators report a perioperative mortality over 60% in comatose patients undergoing surgery for ATAAD (2). As a result, many patients are often deemed inoperable due to indicated poor postoperative outcomes. This case stands out due to the successful surgical procedure in comatose patient, which includes postoperative obstruction of the left coronary ostium and successful ultrasound-guided intervention in the left coronary artery.

## 2 Case description

**Figure 1:**
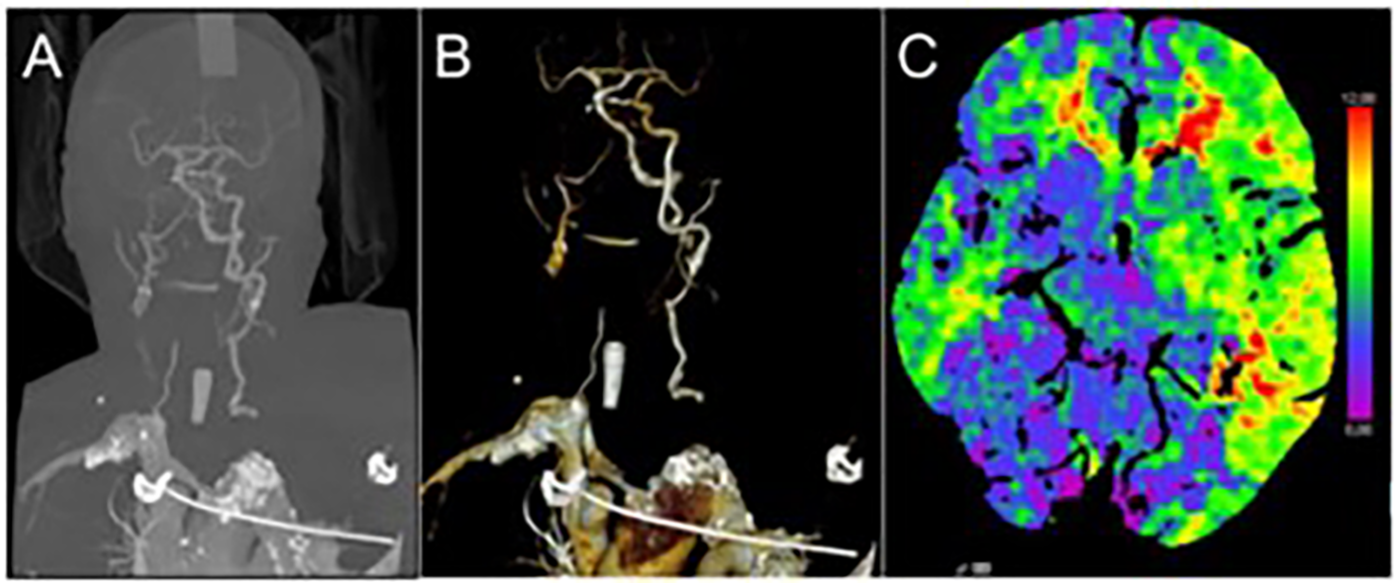
Preoperative contrast-enhanced computed tomography of the supra-aortic rteries showing long-segment antegrade obstruction of both carotid arteries and the right vertebral artery **(A,B)** and cerebral perfusion deficit predominately over the right cerebral hemisphere **(C)**.

A 74-year-old female with a history of arterial hypertension and previous stroke without residual deficits, was admitted to the emergency department. She was found in a public restroom, disoriented, aphasic and left-sided hemiplegic. The initial GCS was 13. After transfer to our stroke unit, a CT scan showed an ATAAD with a large primary entry in the ascending aorta. In addition, long-segment obstructions of both carotid arteries and the right vertebral artery were found, with a remaining cerebral perfusion through the left vertebral artery as the final remaining vessel (Figures 1A,B). This resulted in a pronounced perfusion deficit in the right cerebral hemisphere, with a mismatch of 355 ml (Figure 1C). After arrival, she deteriorated rapidly to a GCS of 3 and was taken straight to the operating theater, which was approximately 3.5 h after symptom onset. The estimated EuroSCORE II value was 58%.

## 3 Timeline

**Figure 2:**
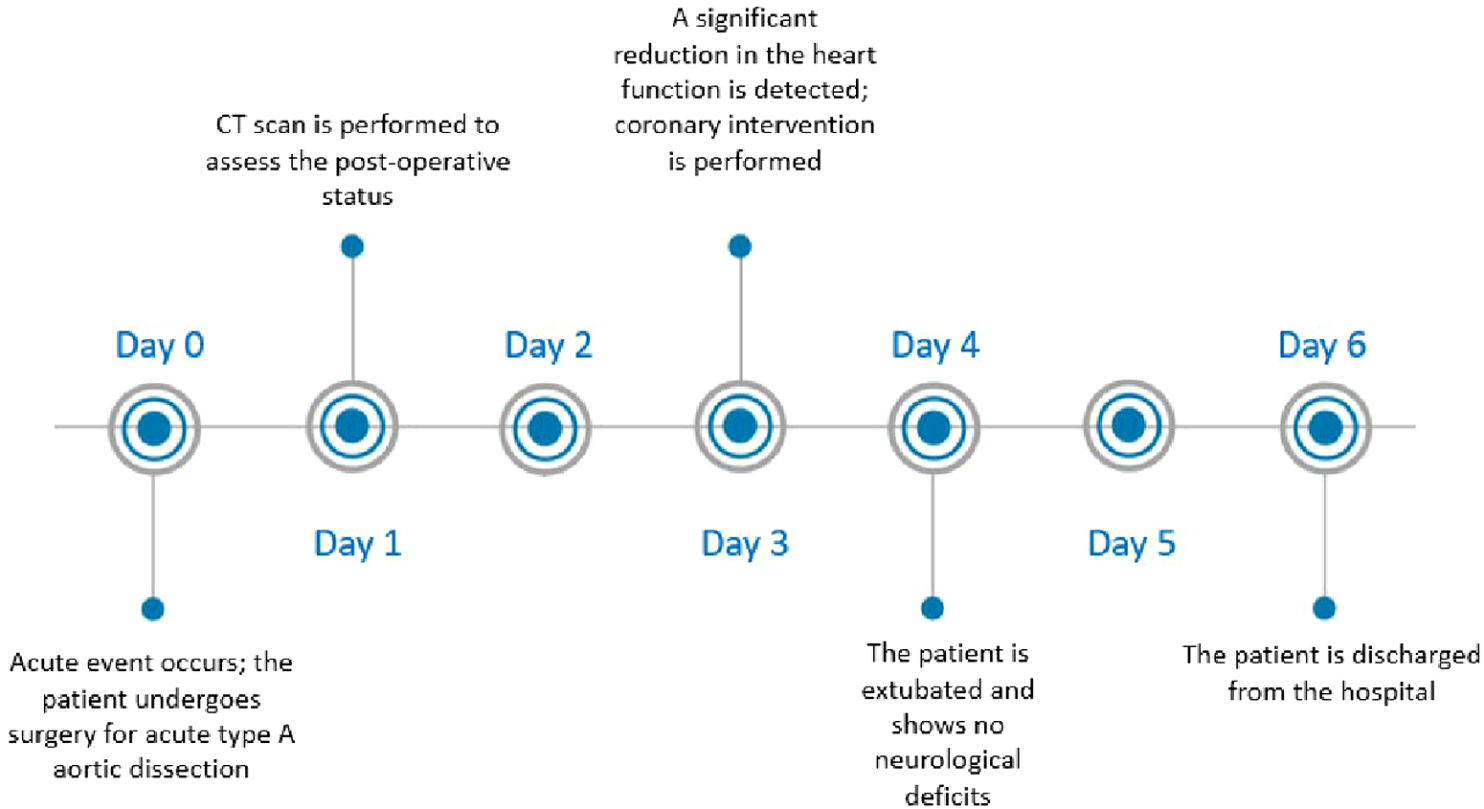
Case report timeline description.

## 4 Diagnostic assessment and treatment

**Figure 3:**
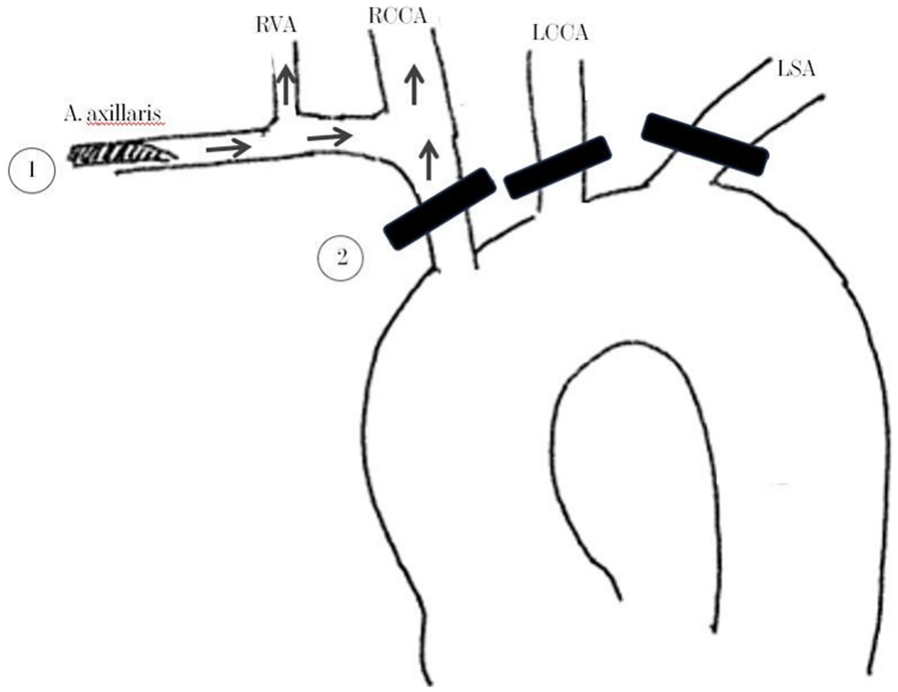
Schematic illustration of the arterial cannulation and the cerebral perfusion technique. (1) Positioning of the 18 French Cannula, (2) Black stripes show the positioning of the double encircled silastic bands “Vessel-loop” for vascular occlusion. Arrows show the direction of blood flow. RVA, right vertebral artery; RCCA, right common carotid artery; LCCA, left common carotid artery; LSA, left subclavian artery.

After rapid-sequence intubation and instillation of a central venous and arterial lines, we cannulated the right axillary artery with an 18 French flexible cannula since this artery was not dissected. Following median sternotomy and pericardiotomy, the right atrial appendage was cannulated with a double-stage venous cannula, and cardiopulmonary bypass was initiated. We aimed for uninterrupted cerebral perfusion for the duration of the operation with antegrade flow via the right axillary artery and the circle of Wills as a primary collateral pathway. The adequacy of the cerebral perfusion (Figure 3) was confirmed by observing an immediate improvement of the Near Infrared Spectroscopy (NIRS) readings over both frontal cortices, which remained stable throughout the surgery.

Systemic cooling was limited to 26°C core temperature to preserve the cerebral autoregulation. The cerebral perfusion pressure was kept between 60 and 70 mmHg, as measured using the left radial arterial catheter. Using the pH-Stat method, moderate hypercapnia was permitted to decrease cerebral vascular resistance and improve cerebral perfusion. Intraoperatively, after clamping the ascending aorta, retrograde cardioplegia was initiated using a 14 French balloon-tipped flexible cannula. Then, the tubular aorta was divided.

The commissures of the aortic valve were exposed and reinforced with pledgeted commissural sutures. No glue or other sealants were used.

After inspecting the coronary ostia, cardioplegia was administered in a selective antegrade fashion using silicon-tipped cannulas. Upon achieving diastolic cardiac arrest, attention was focused on the aortic root, as a target body temperature of 26 °C had not yet been reached. During this time, the tubular part of the ascending aorta was removed, and the aortic root was closely inspected, which in our opinion did not require replacement. Once the target core temperature was achieved, the silastic bands were snared, and unilateral brain perfusion was maintained through the axillary artery in an antegrade fashion via the right vertebral and carotid arteries, without a decrease in NIRS measurements.

Hemiarch replacement with a polyester prosthesis was performed with a 5/0 prolene suture within 9 min of antegrade cerebral perfusion. We documented 32 min of myocardial ischemia and 87 min of cardiopulmonary bypass (CPB). Following the aortic repair, doppler ultrasound of the carotid arteries showed normal antegrade perfusion bilaterally with an adequate NIRS reading. Rewarming, weaning from the heart lung machine, and meticulous hemostasis was achieved.

Postoperatively, the patient was transferred to our intensive care unit (ICU), intubated and remained stable under low-dose vasopressors. The first ECG showed ST segment depressions in I, aVL, and V3–V6, which normalized the following day. The cardiac biomarkers demonstrated a decline in the slope early postoperatively. Partial respiratory insufficiency delayed extubation.

**Figure 4:**
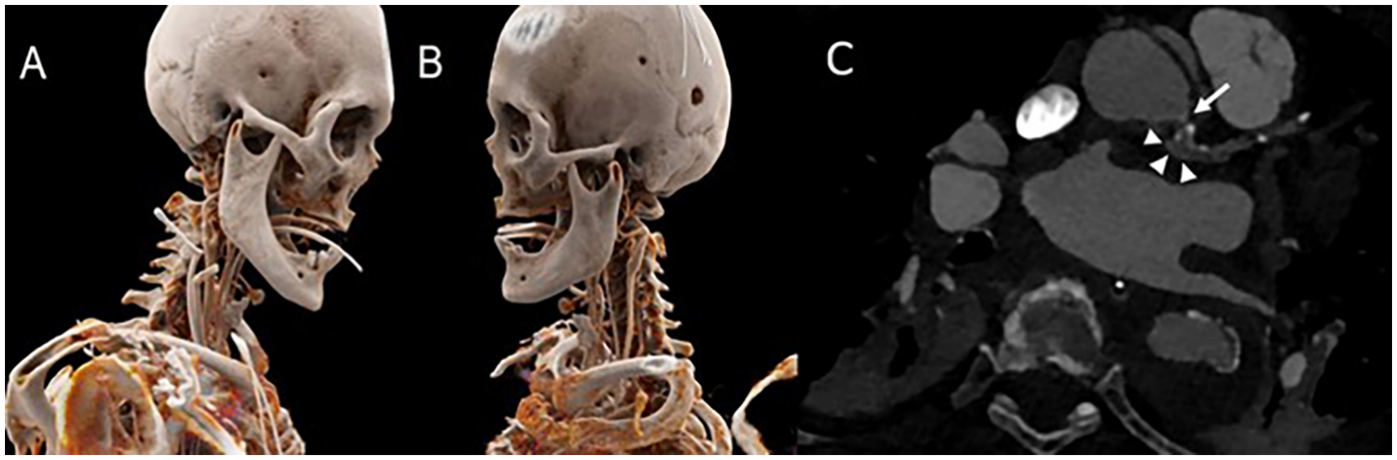
Postoperative CT-scan showing 3-D reconstructions of patient right **(A)** and left **(B)** A CT angiogram displaying a dissection membrane narrowing of the left main ostium (white arrow) and the false lumen (white arrowheads) **(C)**.

A CT angiography showed reestablished normal perfusion of both carotid arteries and a localized dissection of the aortic root with perpetuation into the left main coronary artery (Figure 4). Due to the declining levels of cardiac biomarkers, an unknown neurological outcome, and improvements in hemodynamic stability, no further measures were implemented on the first postoperative day. However, on the third postoperative day new T-wave inversions in leads V3–V6, accompanied by a progressive increase in cardiac biomarkers, were noticed. A transthoracic echocardiographic exam demonstrated a decline in left ventricular ejection fraction from 60% to 25% with global hypokinesia and new moderate mitral valve regurgitation. Because of the rapid hemodynamic deterioration, the only logical explanation for a sudden drop in ejection fracture due to luminal narrowing of the left main ostium. It was presumed that this was most likely because of dynamic changes of the residual dissection in the aortic root or through a hematoma compressing from the outside. Following an interdisciplinary discussion, we decided to proceed with an invasive coronary angiogram, despite the known risks associated with percutaneous coronary intervention (PCI) in cases of coronary dissection, for the following reasons: First, the patient exhibited clear signs of myocardial ischemia. Second, the 90% stenosis of the left main artery posed a significant risk of complete occlusion at any moment, which could result in sudden cardiac death for the patient.

**Figure 5:**
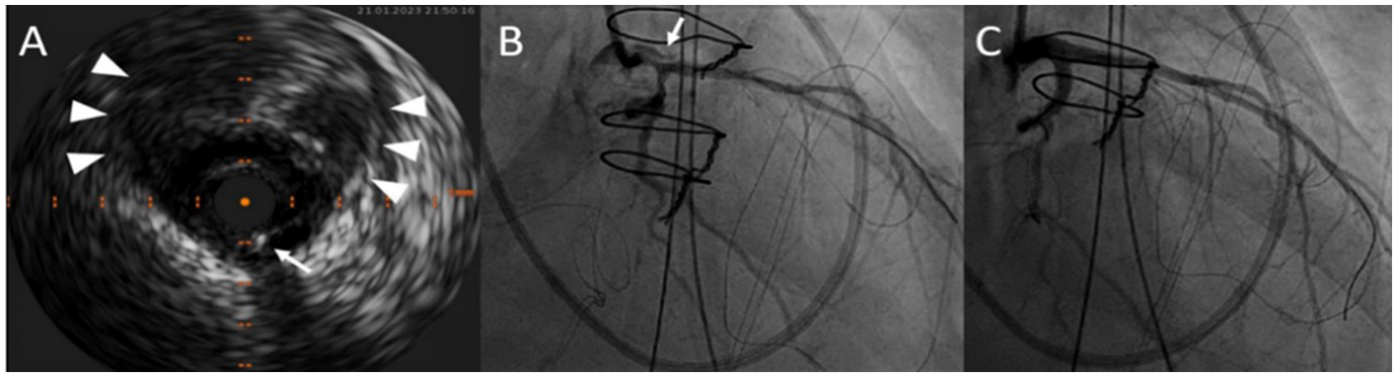
After wiring both the left anterior descending artery and the left circumflex artery, intravascular ultrasound of the left main coronary artery confirmed the correct wire position (white arrows) in the true lumen. The white arrowheads point at the large false lumen **(A)**. RAO projection in the coronary angiogram displaying dissection narrowing of the left main ostium and white arrow points at the false lumen **(B)**. Postoperative result after LAD and LCX stenting with a nano-crush technique **(C)**.

After gaining radial access, a 6 French Judkins left 4.0 guide was advanced to the left main ostium. To prevent the dissection progression, both ostia left anterior descending artery (LAD) and the left circumflex artery (LCX) had to be secured with a stent. The guidewire was advanced in the mentioned arteries without any contrast injection. Correct position of the wires in the true lumen was confirmed by intravascular ultrasound (Figure 5). After wiring the LAD and the LCX, the first contrast injection confirmed the dissection with contrast dye spreading in the false lumen and retrograde towards the aortic root. With no further delay, LAD, and LCX were stented using a nano-crush technique to the edge of the LM ostium without protrusion into the aorta. A Boston Scientific Synergy Megatron 3.5/20 mm stent was used for the LAD, and a Boston Scientific Synergy 3.0/16 mm for the LCX with excellent results. To better assess the result, we performed the IVUS again after stent deployment. The patient was given an antiplatelet therapy with 500 mg Aspirin IV at the start of the procedure and a loading dose of 600 mg Clopidogrel after the PCI. After the intervention, an improvement of the ejection fraction to 40% and a reduction of the residual mitral regurgitation from severe to mild was noticed. The patient was extubated on the fourth postoperative day without neurological impairment. She was transferred to the rehabilitation center on the sixth postoperative day (Figure 2) and returned home on the 42nd postoperative day after she made a full recovery. The patient was maintained on dual antiplatelet therapy consisting of Aspirin and Clopidogrel for 12 months.

## 5 Discussion

ATAAD with a global neurologic deficit is associated with very high mortality and morbidity (1–3). Comatose patients with single-vessel cerebral perfusion very rarely survive the acute event. They are often declared inoperable (4), even though some studies suggest that an early intervention may lead to a postoperative improvement of the neurologic deficit (5–9). Further, the results of the IRAD suggests that an ongoing brain ischemia should not be a contraindication for the surgery, especially if the patient does not display definitive signs of severe irreversible neurological damage (2). The decision to operate our patient was taken after observing short periods of cognitive bursts and alternating neurologic symptoms, which most likely was due to a dynamic low-flow and not a complete interruption of the perfusion to the brain.

It is our belief that cannulating the right axillary artery could be the most expedient method to restore antegrade cerebral perfusion, especially in cases where the dissection membrane occlude the ostia of supra-aortic branches. The real-time NIRS findings supported our judgment, as the regional oxygen saturation improved once the patient was placed on the extracorporeal circulation using axillary cannulation. Additionally, intraoperative carotid Doppler sonography confirmed sustained extracranial perfusion. Nevertheless, it is critical not to overlook the aortic root and coronary ostia after reestablishing brain perfusion, given that coronary involvement in ATAAD significantly predicts both early and late mortality (10, 11).

However, when neurologic symptoms are present without evident myocardial compromise, priority shifts towards re-establishing cerebral perfusion. Unfortunately, the preoperative CT scan did not include the aortic root because of the neurologist's initial focus. During the operation, we did not observe any signs of aortic root dissection, which is why we did not underwent aortic root replacement. In our opinion, the root dissection likely occurred postoperatively, and was subsequently detected in the postoperative CT scan. The weaning of heart lung machine was uneventful, and we saw no significant echocardiographic or ECG changes that would indicate myocardial ischemia due to the left main obstruction. In retrospect, an earlier intervention upon detecting the narrowing of the left main artery would have prevented the subsequent hemodynamic deterioration. However, our approach was aimed at simplifying the post-operative course. The argument stands that intraoperative coronary angiography could offer valuable insights into myocardial perfusion, potentially improving postoperative outcomes without adding procedural complexity (12).

Upon detecting these signs of myocardial ischemia, PCI for the left main dissection was undertaken despite the inherent risks associated with treating coronary dissections. In conclusion, this case underscores the effectiveness of a multidisciplinary approach and feasibility of PCI for left main dissection in a challenging cardiovascular emergency.

## Data Availability

The original contributions presented in the study are included in the article/Supplementary Material, further inquiries can be directed to the corresponding author.
